# A rare cause of recurrent gastrointestinal bleeding: mesenteric hemangioma

**DOI:** 10.1186/1749-7922-4-5

**Published:** 2009-01-29

**Authors:** Mircelal Kazimi, Murat Ulas, Cem Ibis, Mutlu Unver, Nazan Ozsan, Funda Yilmaz, Galip Ersoz, Murat Zeytunlu, Murat Kilic, Ahmet Coker

**Affiliations:** 1 Ege University Medical Faculty Department of General Surgery, Organ Transplantation and Research Center, Izmir, Turkey

## Abstract

Lower gastrointestinal hemorrhage accounts for approximately 20% of gastrointestinal hemorrhage. The most common causes of lower gastrointestinal hemorrhage in adults are diverticular disease, inflammatory bowel disease, benign anorectal diseases, intestinal neoplasias, coagulopathies and arterio-venous malformations. Hemangiomas of gastrointestinal tract are rare. Mesenteric hemangiomas are also extremely rare.

We present a 25-year-old female who was admitted to the emergency room with recurrent lower gastrointestinal bleeding. An intraluminal bleeding mass inside the small intestinal segment was detected during explorative laparotomy as the cause of the recurrent lower gastrointestinal bleeding. After partial resection of small bowel segment, the histopathologic examination revealed a cavernous hemagioma of mesenteric origin.

Although rare, gastrointestinal hemangioma should be thought in differential diagnosis as a cause of recurrent lower gastrointestinal bleeding.

## Introduction

Lower gastrointestinal hemorrhage is defined as an abnormal intraluminal blood loss from a source distal to the ligament of Treitz. Lower gastrointestinal hemorrhage can be due to numerous conditions, including diverticulosis, anorectal diseases, benign or malignant neoplasias, inflammatory bowel disease, and angiodysplasias. Coagulopathies can also be the cause of lower gastrointestinal bleeding. Although hemangiomas can be seen in liver, osseous tissues, mediastinum, soft tissues and other organs, intestinal hemangiomas of mesenteric origin are extremely rare.

We report a case of mesenteric hemangioma of small intestine causing lower gastrointestinal bleeding.

## Case

**Figure 1:**
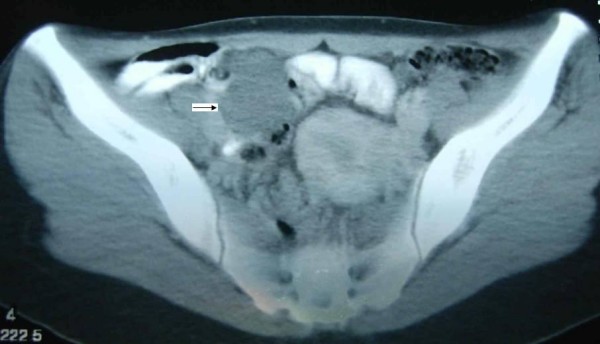
**Oral and intravenous contrast enhanced computed tomography scan showing the mesenteric mass of the ileal small bowel segment (arrow)**.

A 25-year-old female was admitted to the emergency room with fatigue, recurrent black stools. She was hospitalized because of gastrointestinal hemorrhage. Profuse anemia with a hemoglobin level of 4.4 g/dl and the hematocrit 17% was detected. Three packs of red blod cell were transfused immediately. She did not have obvious hematochesia The upper gastrointestinal endoscopy did not show any bleeding lesion. An antral gastritis was only detected during the gastroduodenoscopy. Double contrast barium enema was also normal. We canceled the previously scheduled colonoscopic examination after detecting a 5 × 4 cm sized abdominal mass in the small bowel mesentery through abdominal computed tomography (Figure 1). Surgical exploration was planned. During the explorative laparotomy, a 5 × 5 cm sized mass was detected in the mesentery of the ileum. Partial small bowel resection and end-to-end small bowel anastomosis was performed. She was discharged on the 6th postoperative day. Six months follow-up was uneventful.

**Figure 2:**
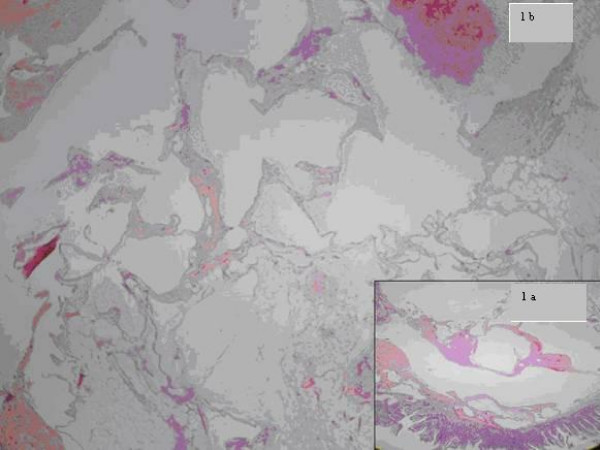
**Mesenteric cavernous hemangioma with thin vascular wall and luminal cystic dilatation (1a-b, H&E, ×2, ×10)**.

**Figure 3:**
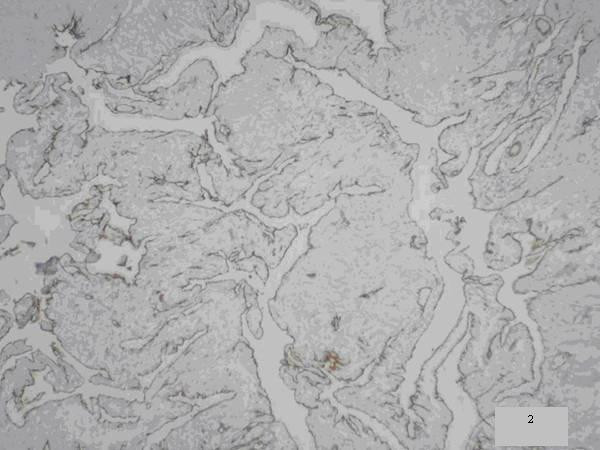
**Immunohistochemical CD31 staining of endothelial cells flooring dilated vessel (2, ×10)**.

Histopathologic examination of the resected specimen revealed a cavernous hemagioma of mesenteric origin (Figures 2, 3).

## Discussion

It is generally believed that hemangioma is a congenital hamartomatous lesion that originates from embryonic sequestrations of mesodermal tissue [1-5]. Hemangioma is a benign tumor, which can be seen in many organs. Approximately 200 cases of gastrointestinal hemangiomas have been reported since 1839 but only a few of these have been reported to involve the mesentery and part of the gut [1]. A classification system used by Abrahamson and Shandling divides intestinal hemangiomas into three categories on the basis of histologic appearances: capillary, cavernous, and mixed type [6]. The most common type is the cavernous hemangioma [6,7]. Cavernous hemangiomas are macroscopically bluish purple, soft and compressible structures, arising from larger submucosal arteries and veins with varying lesion sizes.

Gastrointestinal hemangiomas arise from the submucosal vascular plexuses and may invade the muscularis layer. There is rarely penetration beyond the serosa [10]. Gastrointestinal hemangiomas have been reported in patients ranging from 2 months to 79 years of age. No obvious sex predominance has been identified. They usually present in young men and women, often in the third decade of life [1-3].

The symptoms of hemangioma depends on the localization of the primary tumor. Eighty percent of patients with gastrointestinal hemangiomas presents with symptoms such as bleeding or obstruction [7,8]. The major symptom of gastrointestinal hemangiomas is bleeding [7]. Whereas bleeding from capillary type lesions tends to be slow or may be occult, the hemorrhage in association with a cavernous hemangioma is usually of sudden onset and may present as either hematemesis or melena [7,8]. Our patient has had also recurrent lower gastrointestinal bleeding episodes in her history.

Hemangiomas may result in hemoperitoneum or intestinal obstruction due to the intussusception of the polypoid tumor. Whereas abdominal pain may become the major complaint in these patients, nausea, vomiting, and abdominal distention may also be found [8-11]. The type of treatment depends on the type of lesions, location, extent of involvement, extent of symptoms, and general operability [10,11].

Gastrointestinal hemangiomas of well-defined segment of intestinum are usually suitable for surgical resection at the time of diagnosis [10,11]. Recurrences after resection are rare [10].

Low-dose radiation therapy, cryotheraphy, brachytheraphy, sclerotheraphy or arterial embolization has been used in nonresectable and diffuse hemangiomatosis with limited success [12,13].

Whereas preoperative definitive diagnosis of a mesenteric hemangioma is nearly impossible, oral and intravenous contrast enhanced computed tomography could be helpful in suspecting and localization of such a lesion. Surgical resection of the involved segment remains as the treatment of choice for suitable cases.

As a conclusion, mesenteric hemangioma may be the cause of recurrent lower gastrointestinal bleeding manifested with anemia, and/or episodes of abdominal pain. Although it is very rare, gastrointestinal hemangioma should be kept in mind after eliminating the more common causes of gastrointestinal hemorrhage in differential diagnosis.

## Competing interests

The authors declare that they have no competing interests.

## Authors' contributions

MK, MU and CI planned and wrote the manuscript. CI translated the manuscript to English. MU collected the datas. NO and FY performed the histopathologic evaluation. GE, MZ, MK and AC analyzed the present data and made the revisions.
